# Photophysics of DFHBI bound to RNA aptamer Baby Spinach

**DOI:** 10.1038/s41598-021-85091-y

**Published:** 2021-04-01

**Authors:** Nguyen Thuan Dao, Reinhard Haselsberger, Mai Thu Khuc, Anh Tuân Phan, Alexander A. Voityuk, Maria-Elisabeth Michel-Beyerle

**Affiliations:** 1 School of Physical and Mathematical Sciences, Nanyang Technological University, Singapore 637371, Singapore; 2 Institute of Materials Science, Vietnam Academy of Science and Technology, Hanoi 100000, Vietnam; 3 TUMCREATE, Singapore 138602, Singapore; 4 Institució Catalana de Recerca I Estudis Avancats, 08010 Girona, Spain

**Keywords:** Chemical biology, Molecular biology

## Abstract

The discovery of the GFP-type dye DFHBI that becomes fluorescent upon binding to an RNA aptamer, termed Spinach, led to the development of a variety of fluorogenic RNA systems that enable genetic encoding of living cells. In view of increasing interest in small RNA aptamers and the scarcity of their photophysical characterisation, this paper is a model study on Baby Spinach, a truncated Spinach aptamer with half its sequence. Fluorescence and fluorescence excitation spectra of DFHBI complexes of Spinach and Baby Spinach are known to be similar. Surprisingly, a significant divergence between absorption and fluorescence excitation spectra of the DFHBI/RNA complex was observed on conditions of saturation at large excess of RNA over DFHBI. Since absorption spectra were not reported for any Spinach-type aptamer, this effect is new. Quantitative modelling of the absorption spectrum based on competing dark and fluorescent binding sites could explain it. However, following reasoning of fluorescence lifetimes of bound DFHBI, femtosecond-fluorescence lifetime profiles would be more supportive of the notion that the abnormal absorption spectrum is largely caused by *trans*-isomers formed  within the *cis*-bound DFHBI/RNA complex. Independent of the origin, the unexpected discrepancy between absorption and fluorescence excitation spectra allows for easily accessed screening and insight into the efficiency of a fluorogenic dye/RNA system.

## Introduction

One of the most challenging problems in cell biology is the control of RNA activity by employing genetically encoded fluorescent labels. To this goal the non-toxic, membrane-permeable reporter 3,5-DiFluoro-4-HydroxyBenzylidene Imadazolinone (DFHBI) mimicking the fluorophore of the Green Fluorescent Protein (GFP) has been engineered that is non-fluorescent in solution but emits green fluorescence when bound to the core G-quadruplex of the RNA aptamer Spinach^1,2^. This discovery paved the road for engineering a large variety of fluorogenic RNA aptamers as powerful tools for imaging genetically encoded domains in living cells^3–5^. Apart from poor photo- and low thermal stability, the applicability of the initial Spinach aptamers to living cells^5^ was expected to be limited also by its propensity to misfold. In the meantime, many limitations are removed either by miniaturizing the Spinach aptamer, or optimized superfolding aptamers, or by variation of the small reporter fluorophore^6,7^.

In search for small aptamers that are easier to integrate into arbitrary RNA sequences, the crystal structure of Spinach allowed for a rational reduction by removing non-essential regions of the sequence, thereby miniaturizing Spinach to Baby Spinach^1,5,8,9^ and, the even smaller Broccoli^10–12^ as well as still shorter variants such as Corn^13,14^. Surprisingly, when genetically inserted into ribosomal RNAs, both of the small aptamers, Baby Spinach and Broccoli, have shown superior fluorescence efficiency as compared to the identical constructs under *in-vitro* conditions^8^ and as compared to several full-sequence Spinach aptamers. Thus, the early conjecture^1^ that the compact Baby Spinach, only half as large as the mother Spinach, may reduce live-cell artefacts when fused to ribosomal RNA^8^, gained confidence. Baby Spinach is also resistant to nuclease cleavage and degradation. Moreover, the smaller and more compact Baby Spinach results in higher mechanical stability as compared to a full Spinach sequence^15^. The structure of Baby Spinach in comparison with full sequence Spinach is shown in Fig. S1.

In view of the increasing interest in small RNA aptamers^9^ and the scarcity regarding their photophysical characterization, this paper is a model study on Baby Spinach. Although there is no high-resolution X-ray structure available, identical NMR spectra of Spinach (SP) and Baby Spinach (bSP) in the imino region suggest that the structure of the binding pocket is retained upon miniaturization^4^. Another feature, though under some debate^8^, is the fluorescence yield from bSP aptamers. More recently, the high fluorescence yield reported for the fluorophore-tagged Baby Spinach approaching 95% of the fluorescence intensity of Spinach is under debate^8^.

In this paper, we report on DFHBI/Baby Spinach studied by steady-state absorption and fluorescence spectroscopy complemented by femtosecond time-resolved fluorescence measurements. While quantitative modelling of the absorption spectrum is consistent with competitive binding to a fluorescent and dark RNA aptamer site in the case of bSP, femtosecond lifetime profiles suggest *cis–trans* isomerisation of the DFHBI/bSP complex^16,17^ within the binding pocket as main contribution to the large amplitude of the absorption spectrum on condition of RNA excess over DFHBI and saturation of fluorescence.

## Results

### Absorption and fluorescence spectra of the DFHBI/bSP system

**Figure 1:**
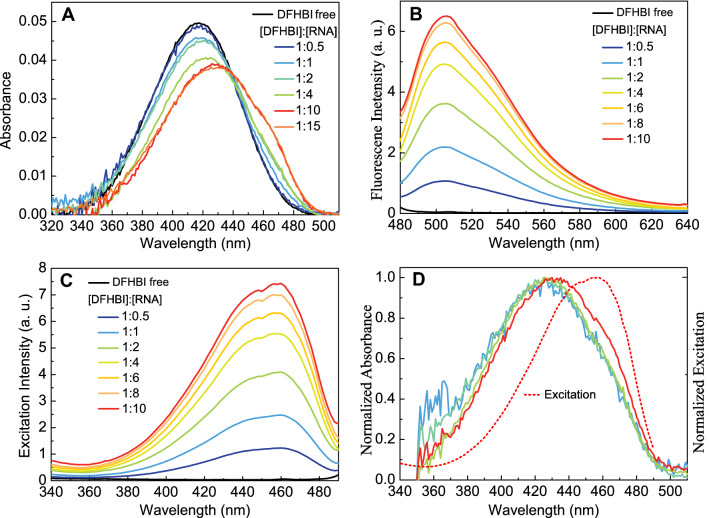
Absorption (**A**) and fluorescence (**B)** spectra (λ_exc_ 460 nm) of free DFHBI (2 µM) in the absence of bSP (black) and its mixture with increasing bSP concentrations, all measured in a flow-cell at low intensity. **(C)** Fluorescence excitation spectra (λ_probe_ 501 nm) of free DFHBI and in the presence of increasing bSP concentrations. **(D)** Normalized calculated absorption spectra for increasing bSP concentrations as derived from K_d_ = 4.4 µM in comparison with the fluorescence excitation spectrum (red, dotted).

*Absorption and Fluorescence.* As shown in Fig. 1A**,** the absorption spectrum of DFHBI anion is strongly affected by increasing amounts of bSP. At saturating bSP concentration the spectrum is broadened, and its peak is red shifted from 416 to 427 nm, while its molar extinction coefficient decreases by ≈ 30% (data in Fig. S2). Such strong hypochromism known to accompany intercalation of dyes between base stacks of DNA is not only consistent with the X-ray structure^5,18^ of the parent SP, but supports also a similar stacking pattern for bSP.

The fluorescence spectrum of the complex DFHBI/bSP (Fig. 1B) is independent of the bSP concentration (Fig. S3). Its peak position is well matched (Fig. S4) with the one observed for DFHBI bound to full-sequence SP^1,5^.

The corresponding fluorescence excitation spectra peaking at 460 nm (Fig. 1C) are blue shifted (9 nm) as compared to the DFHBI/SP complex^2^. In difference to the parent DFHBI/SP complex, excitation and fluorescence spectra of the bSP complex (Fig. S4) are significantly broadened indicating a collection of conformational substates in the complex that seem to depend on preparative details.

#### Determination of the dissociation constant K_d_

The binding affinity of DFHBI (D) to bSP (RNA) can be calculated by applying Eq. (1) to the formation of the fluorescing complex [D•RNA]_FL_ assuming non-cooperative binding (n = 1):

1 $$\left[ {{\text{D}} \bullet {\text{RNA}}} \right]_{{{\text{FL}}}} / \, \left[ {{\text{RNA}}} \right]_{0} = \, \left[ {{\text{D}} \bullet {\text{RNA}}} \right]_{{{\text{FL}}}} / \, \left( {{\text{K}}_{{\text{d}}} + \, \left[ {\text{D}} \right]_{0} - \, \left[ {{\text{D}} \bullet {\text{RNA}}} \right]_{{{\text{FL}}}} } \right)$$

The dissociation constant K_d_ = 4.4 µM has been obtained by fitting fluorescence titration data with Eq. (1). This high K_d_ value is indicative of a relatively low binding efficiency. It is beyond the range of literature K_d_ values^1,16–18^ for the full Spinach sequence, although these are scattered over a wide range between 0.3 μM^16,18^ and 1.3 μM^17^. The scatter depends on details of the preparative protocol and the measuring method used (see SI). Note that these measurements in the literature employed only fluorescence data, and their analysis is based on 1:1 complexation of DFHBI and Spinach RNA.

With the value K_d_ = 4.4 µM obtained from the fluorescence titration, the equilibrium concentrations of the free dye [D] and fluorescent complex [D•RNA]_FL_ were calculated for different ratios of [D] and [RNA]. On the assumption that a 1:1 fluorescent complex is formed, the calculated absorption spectrum of the fluorescent complex in each mixture (Fig. S5) was derived by subtracting the absorption of the free dye from the observed absorption of the mixture according to

2 $${\text{Abs}}_{{[\text{D}\bullet\text{ RNA}]}_{\text{FL}}}\left(\uplambda \right)=\frac{1}{{[\text{D}\bullet\text{ RNA}]}_{\text{FL}}}\left\{{\text{Abs}}_{\text{MIXTURE}}\left(\uplambda \right)-{\text{Abs}}_{\text{D}}\left(\uplambda \right)\right\}$$

where ${\text{Abs}}_{\text{D}}\left(\uplambda \right)$ and ${\text{Abs}}_{\text{MIXTURE}}\left(\uplambda \right)$ are the respective absorption spectra of free DFHBI and its mixture with the aptamer. Figure 1D compares the normalized calculated absorption spectra of the fluorescing complex with the normalized excitation spectrum that is independent of the RNA concentration. Most strikingly, in Fig. 1D there is a large discrepancy between the apparent absorption and excitation spectra of the fluorescent complex independent of the concentration ratio. Thus, the simplest and commonly used binding model cannot be applied to derive the absorption spectrum of the fluorescent complex, and an extended model for binding should be considered. We also noted that even for the Spinach-DFHBI complex, there are large discrepancies in the excitation spectra reported by different groups in the literature (Fig. S6). Although none of these groups reported the absorption spectrum of the complex, the large discrepancy in excitation spectra reported by these groups would clearly modify the discrepancy between absorption and excitation spectra of the Spinach-DFHBI complex.

### Modelling of absorption spectra

In the simplest model, DFHBI forms a fluorescent complex with bSP RNA that can be described by the equilibrium constant K_d_

3 $${\text{K}}_{\text{d}}\text{ = }\frac{\left[{\text{D}}\right]\text{.}\left[{\text{RNA}}\right]}{{\left[\text{D}\bullet\text{RNA}\right]}_{\text{FL}}}$$

Since the fluorescence intensity I_FL_ is proportional to the concentration of the complex: I_FL_ = f [D•RNA]_FL_, Eq. (3) can also be written as

4 $$\frac{{\text{I}}_{\text{FL}}}{\text{f}}\text{K}=\left({\left[\text{D}\right]}_{0}-\frac{{\text{I}}_{\text{FL}}}{\text{f}}\right)\left({\left[\text{RNA}\right]}_{0}-\frac{{\text{I}}_{\text{FL}}}{\text{f}}\right)$$

where [D]_0_ and [RNA]_0_ are the initial concentrations of these components and K_d_ = K. As shown in Fig. 1D, this simple model leads to a significant discrepancy of the absorption and excitation spectra of the fluorescent complex.

What comes to mind first, is that the relatively small bSP RNA may be not perfectly folded. If one assumes that a part of RNA exists in unfolded or misfolded conformations Z which do not form the fluorescent complex, the equilibrium RNA Z, defined by a dissociation constant K_z_, should be considered. In this case Eq. (4) reads

4a $$\frac{{\text{I}}_{\text{FL}}}{\text{f}}\text{K}(1+{\text{K}}_{\text{z}})=\left({\left[\text{D}\right]}_{0}-\frac{{\text{I}}_{\text{FL}}}{\text{f}}\right)\left({\left[\text{RNA}\right]}_{0}-\frac{{\text{I}}_{\text{FL}}}{\text{f}}\right)$$

Denoting K_*_(1 + K_z_) by K_d_ in Eq. (4a), we obtain Eq. (3) implying that K found by fitting of experimental fluorescence data depends on the value of K_z_. More important, however, the factor f remains unchanged. Thus, the ratio [D•RNA]_FL_/[D] does not depend on K_z_. In other words, accounting for the formation of the inactive form Z of RNA aptamer structural diversity will not help to resolve the discrepancy of the absorption and excitation spectra of the fluorescent complex.

#### Multiple-site binding model

Now we consider that in addition to the fluorescent complex also a multitude of non-fluorescent complexes [D•RNA]_DARK_ = [X] is formed. For the sake of simplicity, the multitude of independent binding sites is subsumed by the notion dark complex X. The stability of the dark complex is defined by the dissociation constant

5 $${\text{K}}_{\text{X}}=\frac{\left[\text{D}\right]\left[\text{RNA}\right]}{\left[\text{X}\right]}$$

For convenience, we will use a parameter p = 1 + K_d_/K_x_. Note that p = 1 corresponds to the standard model with a single binding site.

In this extended model, p > 1 and Eq. (4) transforms to Eq. (6)

6 $$\frac{{\text{I}}_{\text{FL}}}{\text{f}}\text{K}=\left({\left[\text{D}\right]}_{0}-\frac{{\text{I}}_{\text{FL}}}{\text{f}}\text{p}\right)\left({\left[\text{RNA}\right]}_{0}-\frac{{\text{I}}_{\text{FL}}}{\text{f}}\text{p}\right)$$

Replacing f by f′/p and K by K′/p in Eq. (4) we obtain

7 $$\frac{{\text{I}}_{\text{FL}}}{\text{f}}{^{\prime}}\text{K}{^{\prime}}=\left({\left[\text{D}\right]}_{0}-\frac{{\text{I}}_{\text{FL}}}{\text{f}}{^{\prime}}\right)\left({\left[\text{RNA}\right]}_{0}-\frac{{\text{I}}_{\text{FL}}}{\text{f}}{^{\prime}}\right)$$

**Figure 2:**
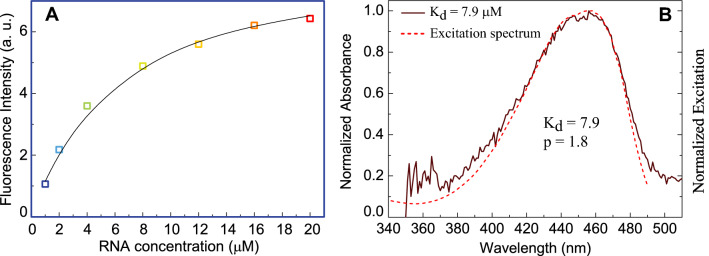
**(A)** Comparison of experimental and calculated fluorescence intensity. The continuous line represents the best fit of the experimental data to the multiple-site model. **(B)** The normalized calculated absorption spectrum of the DFHBI/bSP complex derived with K_d_ = 7.9 µM in comparison to the observed fluorescence excitation spectrum (Fig. 1D).

Equation (7) has the same form as Eq. (4). It means that the dissociation constant and the factor f within the extended model can be directly expressed by the corresponding quantities derived within the standard model, f′ = f _*_ p and K′ = K_*_ p. The concentration of the fluorescent complex is by the factor p smaller than that derived within the simple model, [D•RNA]_FL_ = I_FL_/f′ = I_FL_/(f _*_ p). The factor p cannot be derived from the fluorescence data. However, this parameter can be determined using the absorption spectra shown in Fig. 1A

8 $$\text{p}=\frac{{\text{A}}_{0}^{416}-{\text{A}}_{\text{i}}^{416}}{{\text{A}}_{\text{i}}^{460}}.\frac{{\upepsilon }^{460}}{{\upepsilon }^{416}}$$

where ${\text{A}}_{0}^{416}$ is the absorbance of the pure dye at 416 nm*,* ${\text{A}}_{\text{i}}^{416}$ and ${\text{A}}_{\text{i}}^{460}$ are absorbance of the mixture i (at different concentration ratios) at 416 nm and 460 nm, ε^416^ and ε^460^ are the corresponding molecular extinction coefficients at 416 nm and 460 nm. From the absorption spectra of the mixtures given in Fig. 2A we obtain p = 1.8 ± 0.1. With this value for p and K = 4.4 μM, we obtain a larger dissociation constant K_d_ for the fluorescent complex, K_d_ = K _*_ p = 7.9 ± 0.2 μM than the one extracted from the fluorescence titration. As compared to K_d_ of the fluorescing complex, the dissociation constant K_x_ for the dark complex, K_x_ = K_d_/(p − 1) = 9.8 ± 0.2 μM is only slightly larger.

Figure 2A shows good agreement between experimental data and calculations using the multiple-site binding model. Also using K_d_ = 7.9 µM to derive the absorption spectrum of the fluorescing complex largely removes the discrepancy between absorption and fluorescence excitation spectra in Fig. 1D. Both Fig. 2A,B validate our multiple-site binding model. To our knowledge, it is the first time a discrepancy between the absorption and excitation spectra of the DFHBI/RNA system has been recognized. However, we cannot exclude that this discrepancy is a special feature of the system DFHBI/bSP and less relevant for Spinach-analogue aptamers with higher binding affinity.

The parameter p (p > 1) determines the ratio of the fluorescent to the dark complexes [D•RNA]_FL_/[D•RNA]_DARK_ = K_d_/K_x_ = (p − 1). Therefore, the difference of the free energies of these species can be expressed as

9 $$\Delta = \Delta \text{G}\left({(\text{D}\bullet\text{RNA})}_{\text{FL}}\right)-\Delta \text{G}\left({(\text{D}\bullet\text{RNA})}_{\text{DARK}}\right)=\text{RTln}\left(\text{p}-1\right)$$

while (D•RNA)_FL_ is thermodynamically more stable than X (Δ < 0) when p < 2. By contrast, in systems where p > 2, the formation of the dark states becomes dominating (Δ < 0). For bSP, p = 1.8 yields Δ = -0.17 kcal/mol. Because the ratio [D•RNA]_DARK_/[D•RNA]_FL_ is extremely sensitive to Δ, small changes in the structure or in energy parameters of either RNA or the dye can seriously affect the fluorescent properties of the system.

It is interesting to add that for SP the deletion of both the knob region—a non-complimentary stem in SP—and the U50 nucleotide destroyed the fluorescence^19^. The effect has been attributed to folding problems. This observation is in contrast to the strong fluorescence reported for bSP where also the knob region and the nucleotide U50 are deleted^10^. A direct comparison between the two variants is not possible since the SP mutant^10^ still contains the S3 stem which has been removed in bSP. This comparison demonstrates that a small change in the sequence—even in the region outside of the binding pocket—may lead to a minor change in the structure that can largely affect the fluorescence.

### Quantifying fluorescent complexes and the dark states

**Figure 3:**
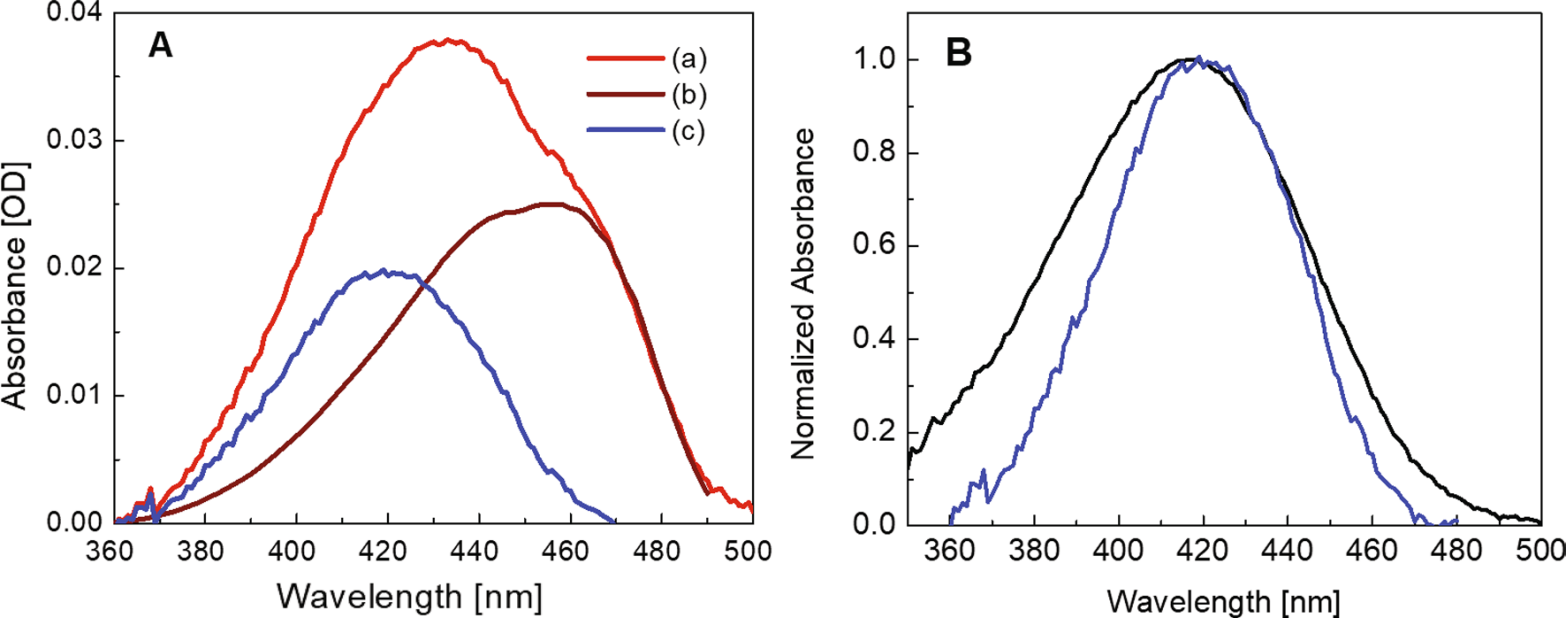
Absorption spectra of the system DFHBI/bSP at saturating conditions 2 µM DFHBI/20 µM bSP. **(A)** **(a)** Measured absorption spectrum of the mixture Fig. 1A (red), **(b)** calculated absorption spectrum of the fluorescing complex (D•RNA)_FL_ (brown), and **(c)** calculated absorption spectrum of free dye and dark complexes (D•RNA)_DARK_ (blue). **(B)** Absorption spectrum of the free dye (black) and absorption spectrum of the mixture of free dye and non-fluorescent complex from **(A)** (blue). Note that the absorption spectrum of the mixture is narrowed and slightly red shifted from 416 to 419 nm.

The absorption spectrum of the mixture 2 µM DFHBI/20 µM RNA comprises two components: the absorption of the fluorescing complex saturated in the presence of 20 µM RNA and the combined absorption of non-fluorescing species consisting of the free dye and the dark complex. The separation of the two components is achieved as follows: Since the absorption spectrum of the fluorescent complex (D•RNA)_FL_ should be identical to the excitation spectrum of its fluorescence at the low concentration of 2 μM used, the combined absorption spectra of the free dye and the dark complex can be constructed for the various DFHBI/bSP ratios as given in Fig. 3A for saturating conditions at a tenfold excess of bSP over the fluorescence reporter DFHBI. (We note in passing that at the low dye concentration used, absorbance measured in OD based on the _10_log scale and the excitation spectrum are similar although the latter based on the number of absorbed photons is directly dependent on concentration.)

The combined absorption spectrum of the free dye and the dark complex (Fig. 3B) is slightly red-shifted peaking at 419 nm and narrower than the absorption spectra of free DFHBI in Fig. 1A. At saturating conditions (2 µM DFHBI/20 µM bSP) and with the dissociation constants for the fluorescing and the dark complex, K_d_ = 7.9 μM and K_x_ = 9.8 μM, respectively, the mixture contains 1.3 μM fluorescing complex, 0.55 μM dark complex, and 0.15 μM free dye. The error bar of these values is about ± 8%.

In the case of bSP, the large dissociation constant of the fluorescing complex indicates poor binding, i. e. a binding efficiency of 65 ± 8% that is similar to the 55 ± 7% binding efficiency reported for DFHBI/bSP when folding was achieved upon heating the RNA aptamer and slow cooling^5^. In the present case, folding was performed at room temperature. It is added in passing that the binding efficiency in Ref.^5^ was estimated using a previously described assay^7^ that is based on the standard 1-site model. However, at the saturating conditions treated, the 1-site standard model and the multiple-site model are equivalent.

### Fluorescence decay kinetics of DFHBI free in solution and in bSP complex

**Figure 4:**
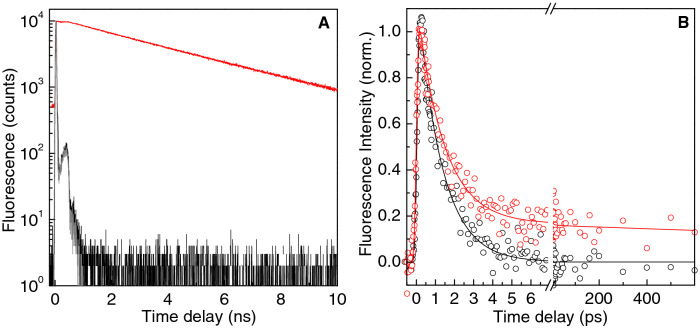
Fluorescence decay probed at 500 nm of DFHBI (2 μM) in absence (black) and presence (red) of bSP (20 μM) under flow cell conditions. (A) Time Correlated Single Photon Counting (TCSPC) measurements of the free dye (black) in solution excited at 416 nm and of the complex excited at 470 nm (red), excitation power 10 μW.The fluorescence decay of the free dye follows the instrument function IRF and is resolved in Fig. 4B. (B) Fluorescence up-conversion measurements of the free DFHBI (black) and the DFHBI/bSP mixture (red), excitation at 400 nm (25 mW).

The excited state of DFHBI in complex with bSP decays almost monoexponentially within 4 ± 0.1 ns (Fig. 4A). This lifetime which has been reported also for the parent DFHBI/SP complex^16^ is indicative of a similar complex in the case of the smaller aptamer complex with bSP.

In Fig. 4B we compare the fluorescence decay patterns of the free DFHBI and of the DFHBI/RNA mixture under saturating conditions. Both measurements were performed in fluorescence up-conversion with a time resolution better than 100 fs in a flow-cell. The fluorescence decay of the free DFHBI could be fitted using a single exponential with a time constant of 1.2 ± 0.02 ps. This time constant is similar the one obtained with higher (20 fs) resolution that showed a biexponential decay giving a mean decay time of 0.97 ps^20^. In analogy to the ~ 1 ps lifetime of the excited GFP chromophore, the ultrashort lifetime of the excited DFHBI is attributed to photoisomerization leading to internal conversion^21,22^.

In contrast to free DFHBI, the fluorescence decay of the DFHBI/bSP mixture fitted to a tri-exponential shows also slower component. First, there is an obvious offset that is constant in the time window (< 1 ns) accessible in femtosecond up-conversion experiments. The offset is caused by the 4 ns lifetime of the fluorescing complex as resolved in the TCSPC measurement (Fig. 4A). In addition to the ~ 1 ps component reflecting the free dye, a second short component of about 10 ps can be fitted, however only with an amplitude of maximal 10% that is too small to account for 28%, dark DFHBI/bSP complexes, as determined in the model of this paper. It should be added that by the nature of fitting and the closeness of the ps time constants, the uncertainties are large. As a consequence, the lifetimes of the free and the dark components cannot be safely discriminated.

## Discussion

How could an ultrashort fluorescence decay time of a bound DFHBI be explained? Following the successful modelling of the steady state absorption spectra (Fig. 3) there may be one or more non-specific binding modes. These may either involve grooves or loops of the bSP structure allowing for hydrogen-bonding and electrostatic interactions in the dark bound states.

Apart from the unknown identity of the dark complexes, also the nature of fluorescence quenching is of interest. In principle, mechanisms that come to mind are photoinduced electron transfers preferentially involving guanine, and internal conversion enabled by almost full rotational freedom of DFHBI in the unspecified dark binding sites. A priori, oxidative electron transfer seems to be improbable considering the anionic state of DFHBI and the large coupling needed to satisfy the fast 10 ps rate in a non-stacked structure. Alternatively, there are a few arguments supportive of short lifetimes of a weakly bound complex. (i) It could be that H-bonding of the dark complex accompanied by some bending of the exocyclic bridge in the DFHBI fluorophore already approaches the conical intersection and it needs only minute rotational motion to cross over to the electronic ground state. (ii) If we translate features of the GFP chromophore to the fluorinated analogue DFHBI where the same methyne-group bridges the phenol and the heterocycle, the underlying photoisomerization may also in DFHBI be a volume conserving process^23,24^ that is unlikely to involve a large scale structural rearrangement and that may therefore be fast. (iii) Arguing on the level of absorption spectra, quantum chemical calculations show (SI) that H-bonding of DFHBI to the neighbouring guanine G31 has little effect on the absorption spectrum. H-bonding, however, may well be the predominant binding mode of a multitude of the complexes. This speculation could gain support by the similarity of the combined absorption spectra of the free DFHBI and the dark complex (Fig. 3B) showing that the absorption maxima are by-and-large overlapping although the contribution of the dark complex is predominant.

However, the very same spectral argument holds also for the assumed overlapping of cis- and trans-conformer of DFHBI. Although all these considerations can support non-specific H-bonding of DFHBI associated with fast fluorescence decay, so far no argument is compelling enough to devalue the observation that the fluorescence amplitude of the apparent dark complex is far too small to explain the large contribution (28%) of the apparent dark state to the absorption spectrum.

In this situation we resort to a molecular specificity of DFHBI. As revealed by X-ray structural analysis^5,18^ of the DFHBI/Spinach complex, DFHBI is sandwiched between the upper G tetrad of a G-quadruplex stretch and the triple bases (UAU, see Fig. S7). The pocket binds the planar cis-isomer of DFHBI by π-π stacking to both, the quadruplex platform and the UAU cap and allows, in addition, for hydrogen bonding of DFHBI to the co-planar guanine (G31). Most importantly, binding of the DFHBI anion in the RNA pocket has been shown not to prohibit photo-isomerisation and escape of the non-planar trans-conformer into the solution^16,17^. Although this loss is reversible, it is the origin of the photochemical instability that is particularly harmful for imaging experiments.

In the experiments of this paper, the accumulation of non-fluorescent trans-isomer of DFHBI bound to Baby Spinach is at least partially suppressed by performing all steady state measurements at lowest possible light intensity and in a flow-cell. Experiments on kinetics of the DFHBI/bSP system in order to quantitatively assess contributions of the trans-conformer to the absorption spectrum together with kinetic modelling are in progress.

In parallel, the suggestion that the accumulation of *trans*-conformers with short lifetimes is at least partially responsible for the discrepancy of absorption and excitation spectra should certainly be tested on its general validity in experiments spanning the role of preparative details and truncated vs full sequence of Spinach, sequence as well as on other miniaturized aptamers as Broccoli^10–12^ and Corn^13,14^. Another attractive line of experiments this paper might stimulate is along the recent developments of modified DFHBI fluorophores that carry bulky substituents^25^ for slowing down internal rotation and thus *cis–trans* isomerisation. Finally, addressing again the present issue of absorption vs. fluorescence excitation spectra. Recently a synthetic dye termed HPC has been reported^26^ that maintains the core structure of the GFP chromophore. HPC is non-fluorescent in solution but shows enhanced fluorescence by more than a factor 3000 when expressed in *E. coli* cells and also in vitro upon binding to the miniaturized RNA aptamer Pepper530. Pepper530 in complex with HBC showed high stability, i.e. a dissociation constant K_d_ = 3.5 nM that is not yet understood in structural and photophysical terms. However, most relevant in the context of the present paper is that Pepper530 folds independently of the potassium concentration suggesting that it does not contain a G-quadruplex and, in contrast to DFHBI/bSP, absorption and fluorescence excitation spectra are perfectly overlapping as demonstrated in Fig. S8.

In conclusion, the entire field of encoding RNA aptamers for live cell imaging will profit from new approaches and improved insights into mechanistic details of reporter/RNA systems as impressively demonstrated in a recent study of viral RNA dynamics^11,12^. A more general approach to treat these experimental results and remove some of the uncertainties has been developed and will be published elsewhere.

## Materials and methods

### The fluorophore DFHBI and Baby Spinach RNA

*DFHBI* (Lucerna Technologies, USA) was dissolved in DMSO to get 40 mM stock solution and stored at − 20 °C. From that, each stock of diluted solution (200 µM) is made in HEPES buffer (10 mM pH 7.5) containing DEPC treated water, 50 mM KCl and 5 mM MgCl_2_.

#### Baby spinach RNA

RNA (sequence: 5′- GGU GAA GGA CGG GUC CAG UAG UUC GCU ACU GUU GAG UAG AGU GUG AGC UCC -3′) was purchased from Dharmacon Inc in protected form. It was then de-protected following Dharmacon’s protocol using de-protection buffer, purified by HPLC, and lyophilized to dryness. The dry pellet was stored at − 80 °C and before use, suspended in HEPES buffer (10 mM pH 7.5) containing DEPC treated water, 50 mM KCl and 5 mM MgCl_2_. The 2-ml Eppendorf tube containing RNA in buffer was put in a water bath (2 L), then heated to 65 °C for 3 min and left cooling overnight to room temperature to let the RNA slowly fold to the correct conformation. The tube was then stored at -30 °C fridge and used as RNA stock after thawing to room temperature. The integrity of the RNA was confirmed by gel electrophoresis experiment as shown in Fig. S9.

#### The mixture of the DFHBI and Baby Spinach RNA

DFHBI at 200 µM stock was diluted in HEPES buffer (10 mM pH 7.5) containing DEPC treated water, 50 mM KCl and 5 mM MgCl_2_ to get 2 µM DFHBI solution, which contains less than 0.1% DMSO. RNA aliquots were added from the RNA stock to the 2 µM DFHBI solution to achieve different ratios of RNA to DFHBI concentrations in the mixture. The mixture was vortexed for 1 min and left in the dark for 2 h for completion of the binding process. Spectroscopic experiments were carried out right after that. The formation and the integrity of the G-quadruplex binding pocket before and after forming the complex was confirmed by CD spectra as shown in Fig. S10.

#### Suppression of bleaching by using a flow-cell

All our measurements were performed under flow-cell conditions to minimize photo-bleaching unless otherwise stated. A total of 20 ml of the DFHBI/RNA mixture (at different ratios) was prepared in HEPES buffer (10 mM pH 7.5) containing DEPC treated water, 50 mM KCl and 5 mM MgCl_2_. The solution was circulated between a 20-ml bottle and a flow-cell quartz cuvette by a Gilson Minipulse 3 peristaltic pump with a rotation speed of 48 rpm. Fig. S11 clearly shows photostability of bound DFHBI by using the flow-cell setup for recording fluorescence upon excitation at 460 nm with an irradiation power of 650 µW at an averaged photon flux density of 10^14^ photons/mm^2^s.

#### RNA folding efficiency

To determine the folding efficiency of the RNA, we employed a method which was reported in Folding assay section in Online Methods in Ref.^6^. That method compared fluorescence (excited at 469 nm) of the mixture under 2 extreme conditions: one in which the RNA is in excess relative to the DFHBI (0.1 µM DFHBI + 10 µM RNA), and one in which the DFHBI is in excess relative to the RNA (10 µM DFHBI + 0.1 µM RNA). For each condition, the signal from DFHBI without RNA was subtracted from each signal. The signal from the first condition (limiting RNA) was divided by the signal from the second condition (limiting dye) to determine the fraction folded. From Fig. S12, the fraction of properly folded RNA is estimated to be 91%. Identical CD spectra of the Baby Spinach before and after adding DFHBI (Fig. S10) confirmed the formation and the integrity of the G-quadruplex binding pocket before and after forming the complex.

### Absorption and fluorescence measurements. Quantum-chemical calculations

#### Absorption measurements

Absorption measurements were performed on a Varian CARY-100 spectrophotometer at room temperature using a 10 × 2 mm quartz cuvette with 10-mm path length. At the low light intensity (7.1 × 10^10^ photons/mm^2^ s) used in our absorption measurements, no photo-bleaching has been observed.

#### Fluorescence and fluorescence excitation spectra

Fluorescence and fluorescence excitation spectra were recorded on a Jobin-Yvon-Spex Fluorolog3-11 fluorometer using a 10 × 1 mm quartz cuvette in front-face configuration at high RNA concentration or a 10 × 2 mm quartz cuvette with 10-mm path length in right angle configuration for low RNA concentration. All fluorescence spectra are scanned with an integration time of 0.5 s, an excitation and emission slit width of 2 nm, and step size of 1 nm. In all cases, for the fluorescence spectra three continuous scans were averaged.

#### Time-correlated single photon counting (TCSPC)

The time-resolved fluorescence decay with time constants > 15 ps was measured using a-time-correlated single photon counting (TCSPC) set-up from PicoQuant as described elsewhere^27^. Briefly, the output of a Titan:Sapphire Laser (780–1000 nm, 80 MHz, 100 fs) was frequency doubled (SHG) to obtain a 416 (469)-nm excitation and focused onto the sample (average power ~ 10 µW). A portion of the excitation light was used as the start signal for the measurement cycle controlled by a histogram accumulating real-time processor (PicoHarp 300). A time-resolving spectrometer FluoTime 200 with wavelength resolution of 1 nm or better was used to collect the fluorescence signal. This signal was then recorded by a Multi-Channel-Plate Photomultiplier Tube (MCP-PMT) with an overall IRF (Instrument Response Function) FWHM of 40 ps. The samples were held in a quartz flow cuvette (1 mm).

#### Fluorescence up-conversion

Femtosecond time-resolved fluorescence was measured using our fluorescence up-conversion spectrometer (FOG100, CDP). The samples were excited with the second harmonic of a titanium-sapphire laser (Chameleon, Coherent Inc.) at 400 nm (100 fs, 80 MHz). The fundamental beam of the laser at 800 nm travelled through an optical delay line. The fluorescence of the samples was collected and focused onto a 1 mm BBO crystal together with the delayed fundamental beam. The sum frequency (upconverted fluorescence) beam was focused into the entrance of a double-monochromator. The samples were held in a 1 mm quartz flow cuvette.

To ensure photostability all steady-state and time-resolved fluorescence measurements were performed in a flow-cell (Fig. S11).

#### Quantum chemical calculations

For all calculation the program Gaussian 09 (rev. E01)^28^ was used. Vertical excitation energies were calculated using TDA formalism^29^ with the long-range corrected functional CAM-B3LYP^30^. The standard 6-31G* basis set was used. The equilibrium and non-equilibrium solvation energy in a medium with dielectric constant ε was estimated using a COSMO-like polarizable continuum model in the monopole approximation^31^.

## Supplementary Information

Supplementary Information.
