# Supplementary material for: Photophysics of DFHBI bound to RNA aptamer Baby Spinach
Source: Sci Rep. 2021 Apr 1;11:7356. doi: 10.1038/s41598-021-85091-y (PMC8016939; doi:10.1038/s41598-021-85091-y)
Supplement: Supplementary file 1 — Supplementary Information. [file 41598_2021_85091_MOESM1_ESM.pdf]

Photophysics of DFHBI bound to RNA aptamer Baby Spinach

Nguyen Thuan Dao[1,2,*,+], Reinhard Haselsberger[1,3,+], Mai Thu Khuc[1], Anh Tuân Phan[1], Alexander A. Voityuk[4] & Maria-Elisabeth Michel-Beyerle[1,3,*]

[1]School of Physical and Mathematical Sciences, Nanyang Technological University, Singapore, [2]Institute of Materials Science, Vietnam Academy of Science and Technology, [3]TUMCREATE Singapore, [4]Institució Catalana de Recerca i Estudis Avancats, Girona, 08010 Spain,

*corresponding authors
N. T. Dao thuandn@ims.vast.ac.vn
M.-E. Michel-Beyerle mariaelisabeth@ntu.edu.sg

+These authors contributed equally to this work.

Supplementary Information

Treatment of the fluorimetric data. General remarks

Taking into account that $F_{max} \approx f * [D]_0$, equation (1) can be rewritten as

$$F = F_{max} * [RNA]_0 / (K_d + [RNA]_0 + ([D]_0 - [D•RNA]_{FL}) \qquad (1a)$$

In almost all applications that rest on fluorescence increase of dyes upon binding to aptamers the concentration of the free non-fluorescent dye, ($[D]_0$ - $[D•RNA]_{FL}$), is much less than the initial concentration of the free RNA, $[RNA]_0$, and may be neglected. Thus, the dissociation constant K_d is usually derived from fluorometric titrations using the Hill equation

$$F = F_{max} * [RNA]_0 / (K_d + [RNA]_0) \qquad 1b)$$

Paige et al. reported K_d = 537 nM by measuring the increase in fluorescence as a function of increasing chromophore concentration in the presence of a fixed concentration of RNA using a model for 1:1 complexation.

Han et al. and Huang et al. measured similar values for K_d = 390 nM and K_d = 300 ± 68 nM, respectively, Han et al. exploiting binding-unbinding fluorescence kinetics at low DFHBI concentration (< 1 µM).

Wang et al. reported K_d = 1.3 µM using stopped-flow and thermodynamic titration. Fluorescence at a low chromophore concentration (0.1 µM) was related to increasing

concentrations of RNA up to saturation at 20 μM. This condition is similar to the one employed in this paper. We used the more accurate equation (1) applicable to all samples independent of the ratio of DFHBI and RNA.

Paige, J. S., Wu, K. Y. & Jaffrey, S. R. RNA mimics of green fluorescent protein. *Science* **333**, 642-646 (2011).

Han, K. Y., Leslie, B. J., Fei, J. Y., Zhang, J. C. & Ha, T. Understanding the photophysics of the Spinach-DFHBI RNA aptamer-fluorogen complex to improve live-cell RNA imaging. *J. Am. Chem. Soc*. **135**, 19033-19038 (2013).

Wang, P. C., Querard, J., Maurin, S., Nath, S. S., Le Saux, T., Gautier, A. & Jullien, L. Photochemical properties of Spinach and its use in selective imaging. *Chem. Sci.* **4**, 2865-2873 (2013).

Huang, H., Suslov, N. B., Li, N.-S., Shelke, S. A., Evans, M. E., Koldobskaya, Y., Rice, P. A. & Piccirilli, J. A. A G-quadruplex–containing RNA activates fluorescence in a GFP-like fluorophore. *Nat. Chem. Biol.* **10**, 686-691 (2014).

Differences in sample preparation between various groups working on Spinach DNA. Remarks

a) Paige et al. in 2011, only studied full Spinach sequence, used SELEX. They prepared an affinity matrix and attached NHS-activated Sepharose (Agarose resin) to the fluorophore. Then an RNA library was prepared and performed the SELEX. Before mixing with the fluorophore, the RNA was first heated to 75°C and quickly cooled by placing on ice, and then incubated for 30 min with 500 μl of fluorophore resin and washed with 1 ml selection buffer.

b) Wang et al. in 2013, synthesized the RNA by in vitro transcription. They used HEPES buffer (pH 7.4, 40 mM HEPES, 125 mM KCl, 5 mM $MgCl_2$, 1x salmon sperm DNA). DFHBI solutions were diluted from a 20 mM stock in DMSO. Solutions containing DFHBI and Spinach RNA was mixed at room temperature using a RX2000 rapid kinetic stopped flow accessory (Applied Photophysics, Leatherhead, UK). There is no RNA heating or folding was descried in this work.

c) Warner et al. in 2014, synthesized the RNA by in vitro transcription from PCR templates. First, the RNA was heated to 95°C for 2 minutes and cooled on ice for 2 minutes (quick), then the DFHBI was added. After heating to 65°C for 5 minutes, the mixture was cooled to 25°C at the rate of 0.1°C/s (slow cooling).

d) Zhang et al. in 2015 synthesized the RNA by in vitro transcription using a T7 kit. The transcribed RNA was buffer-exchanged twice with a P-6 micro bio-spin column (Bio-Rad) into RNA storage buffer (10 mM Tris acetate (pH 8.0), 0.1 mM EDTA and 10 mM KCl) to remove unreacted nucleotides. The RNA was folded in a selection buffer (40 mM K-HEPES (pH 7.5) and 125mM KCl) by incubation at 90 °C in water

bath for 2 min, followed by slow cooling down to 65 °C, and then 5 mM $MgCl_2$ was added to assist RNA folding and further cooling down to room temperature.

e) Okuda et al. in 2017 synthesized the RNA by in vitro transcription using a T7 RNA polymerase. First, they used snap-cooled to refold the BabySpinach: heating at 90°C for 2 minutes and placing on ice for 2 minutes. Then the DHBI was added, and the mixture was heated to 65°C for 5 minutes and slow cooling at the rate of 0.3°C/s for 20 minutes to 25°C. His method was very similar to Warner et al., 2014 's method, but his slow cooling was still faster than Warner.

f) Our preparation: First, BabySpinach was heated to 65 °C for 3 minutes and left cooling overnight to room temperature (slow). After that, the DFHBI dye was added to form a mixture, then vortexing for 1 minute and incubating in the dark for 2 hours at room temperature. Our buffer is 10 mM HEPES (pH 7.5), containing DEPC treated water, 50 mM KCl and 5 mM $MgCl_2$. Our method was closer to the biological condition since we did the mixing and formed the complex at room temperature.

Paige, J. S., Wu, K. Y. & Jaffrey, S. R. RNA mimics of green fluorescent protein. *Science* **333**, 642-646 (2011).

Wang, P. C., Querard, J., Maurin, S., Nath, S. S., Le Saux, T., Gautier, A. & Jullien, L. Photochemical properties of Spinach and its use in selective imaging. *Chem. Sci.* **4**, 2865-2873 (2013).

Warner, K. D., Chen, M. C., Song, W. J., Strack, R. L., Thorn, A., Jaffrey, S. R. & Ferré-D'Amaré, A. R. Structural basis for activity of highly efficient RNA mimics of green fluorescent protein. *Nat. Struct. Mol. Biol*. **21,** 658-663 (2014).

Zhang, J., et al., Tandem Spinach Array for mRNA Imaging in Living Bacterial Cells. *Sci. Rep.* **5**:17295 (2015).

Okuda, M., Fourmy, D. & Yoshizawa, S. Use of Baby Spinach and Broccoli for imaging of structured cellular RNAs. *Nucleic Acids Res.* **45**, 1404-1415 (2017).

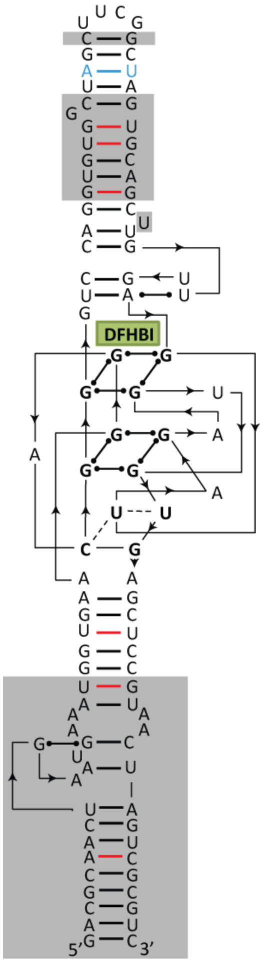

Figure S1. Structure of full sequence Spinach and Baby Spinach aptamer. DFHBI is shown as a green box. Grey boxes are the bases deleted in the Baby Spinach as compared with full sequence Spinach. Blue bases are the inserted mutations presented only in Baby Spinach. Non-Watson-Crick base pairings are colored in red. The sequence of these RNAs was taken from Okuda et al. in 2017 and listed in the table below:

Sequence of Baby Spinach Aptamer[8]

Name	Sequence (5' to 3')
Spinach<br>(98 nts)	GACGCAACUGAAUGAAAUGGUGAAGGACGGGUCCAGGUGUGGCUGCUUCGGC<br>AGUGCAGCUUGUUGAGUAGAGUGUGAGCUCCGUAACUAGUCGCGUC
Baby Spinach<br>(51 nts)	GGUGAAGGACGGGUCCAGUAGUUCGCUACUGUUGAGUAGAGUGUGAGCUCC

Grey bases are Spinach bases deleted in Baby Spinach, blue bases are mutations presented only in Baby Spinach. Color coded the same as Scheme 1.

Okuda, M., Fourmy, D. & Yoshizawa, S. Use of Baby Spinach and Broccoli for imaging of structured cellular RNAs. *Nucleic Acids Res.* **45**, 1404-1415 (2017).

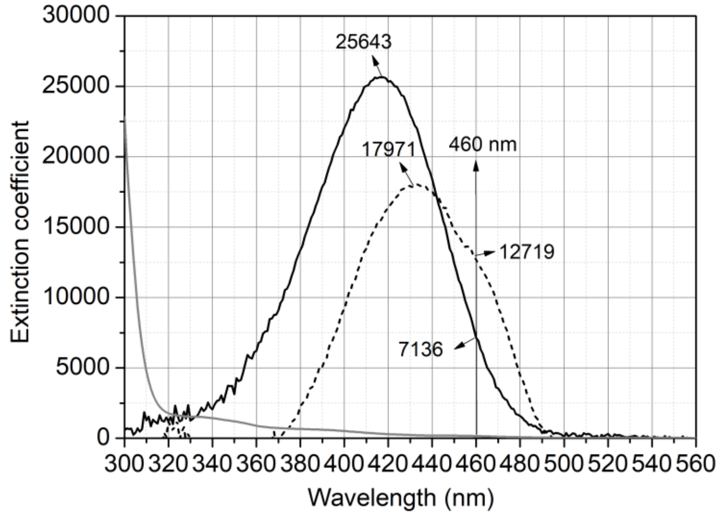

Figure S2. Molar extinction coefficients of the free DFHBI (continuous curve), DFHBI at saturating bSP concentration (dotted curve), and bare bSP (grey curve).

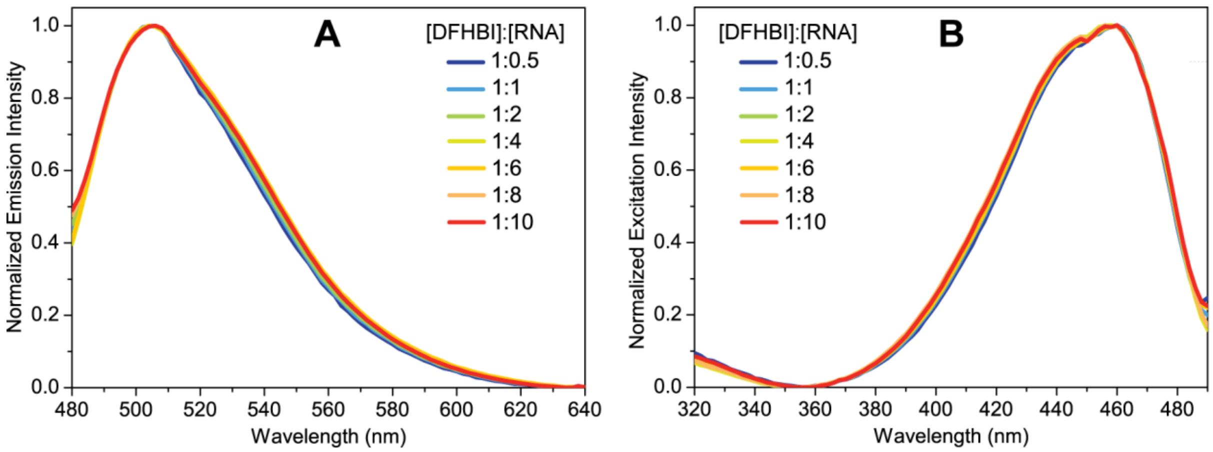

Figure S3. (A) Fluorescence and (B) excitation spectra of the mixture of 2 μM of DFHBI with increasing concentrations of bSP.

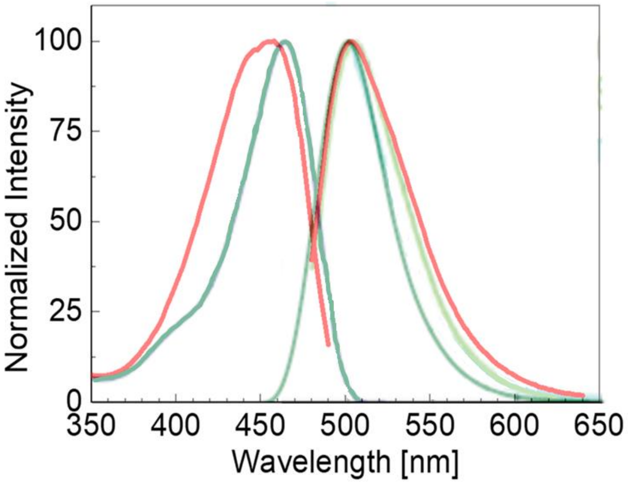

Figure S4. Comparison of experimental excitation and fluorescence spectra. Left: Excitation spectra of DFHBI/bSP (this work, red) and DFHBI/SP (Paige et al., cyan). Right: Fluorescence spectra of DFHBI/bSP (this work, red), DFHBI/SP (Paige et al., cyan), and DFHBI/bSP (Warner et al., green).

Paige, J. S., Wu, K. Y. & Jaffrey, S. R. RNA mimics of green fluorescent protein. *Science* **333**, 642-646 (2011).

Warner, K. D., Chen, M. C., Song, W. J., Strack, R. L., Thorn, A., Jaffrey, S. R. & Ferré-D'Amaré, A. R. Structural basis for activity of highly efficient RNA mimics of green fluorescent protein. *Nat. Struct. Mol. Biol*. **21,** 658-663 (2014).

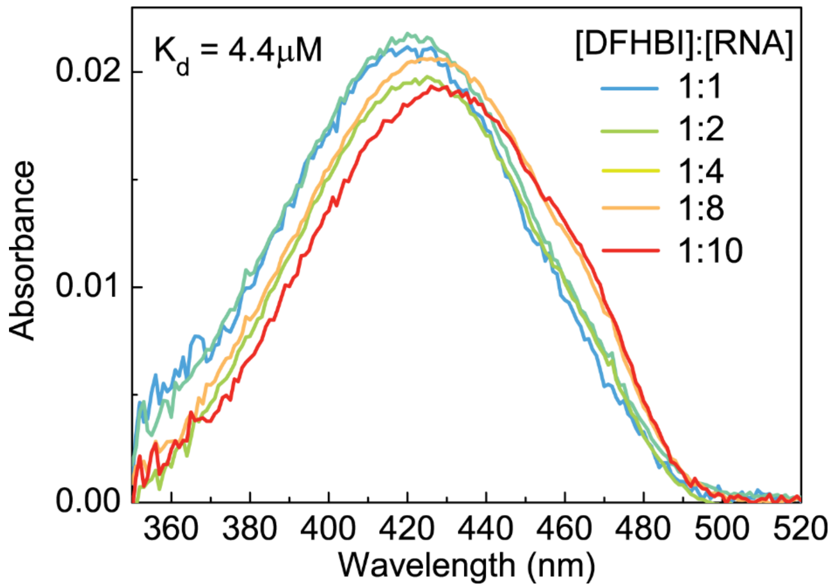

Figure S5. Absorption spectra of the fluorescing complex (normalized to 1 μM complex) derived from experimental absorption spectra using K_d = 4.4 μM and equation (2).

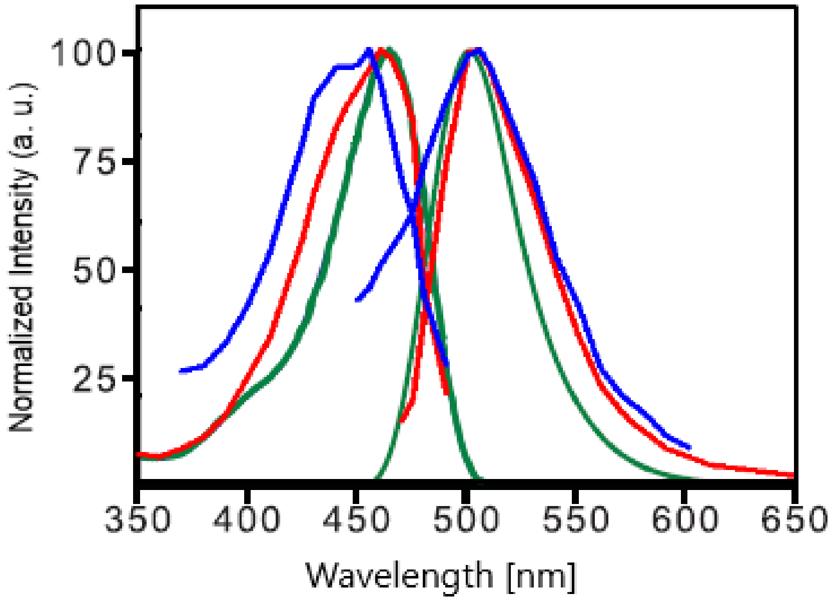

Figure S6. Norm. Fluorescence and fluorescence excitation spectra of DFHBI/SP complex taken from Paige et al. 2011 (figure 3a, green), Han et al. 2013 (figure S3, red), and Okuda et al. 2017 (figure S6, blue).

Paige, J. S., Wu, K. Y. & Jaffrey, S. R. RNA mimics of green fluorescent protein. *Science* **333**, 642-646 (2011).

Han, K. Y., Leslie, B. J., Fei, J. Y., Zhang, J. C. & Ha, T. Understanding the photophysics of the Spinach-DFHBI RNA aptamer-fluorogen complex to improve live-cell RNA imaging. *J. Am. Chem. Soc*. **135**, 19033-19038 (2013).

Okuda, M., Fourmy, D. & Yoshizawa, S. Use of Baby Spinach and Broccoli for imaging of structured cellular RNAs. *Nucleic Acids Res.* **45**, 1404-1415 (2017).

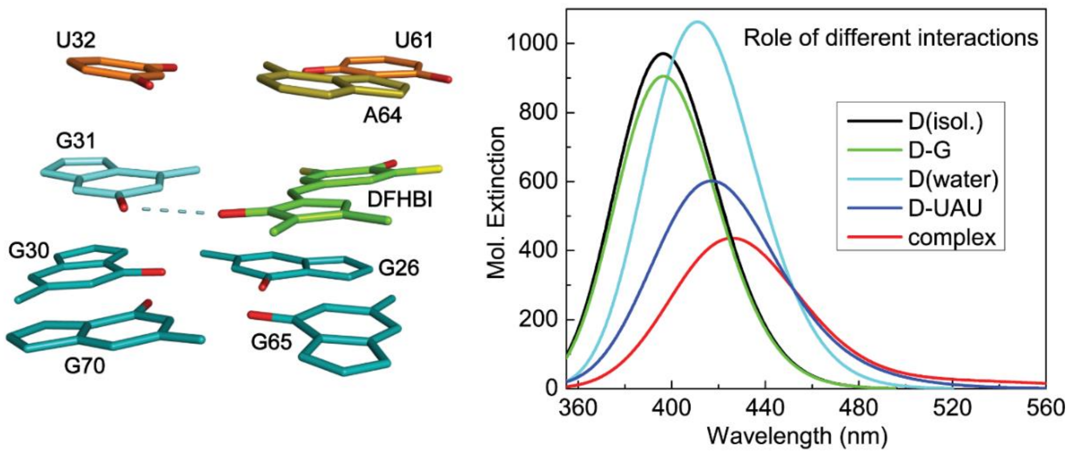

Figure S7. Side-view of a binding pocket showing the DFHBI chromophore and its neighbouring nucleobases (taken from the X-ray structure of Spinach (Warner et al. 2014) and absorption spectra computed for different interactions of DFHBI in the binding pocket. D(isol.) stands for isolated DFHBI alone, D-G for H-bonding of DFHBI to the coplanar guanine G31, D-UAU for π-stacking of DFHBI to the capping nucleobases, D(water) for DFHBI in water environment including H-bonding, and complex for the π-stacking of DFHBI to both, the G-quadruplex platform and the cap.

For a crude insight into the relative effects of different binding interactions of DFHBI on the absorption spectrum of the fluorescing complex, we carried out TDA-DFT (Tamm-Dancoff Approximation Density Function Theory, Hirata et al. 1999) calculations on the components of the model system as shown in Fig. S6. The minimized binding pocket has been constructed on the basis of the X-ray data of RNA Spinach (pdb code **4TS2**) (Warner et al. 2014). There, the G-quartet together with the cap U61, A64, and U32 offers a platform for π stacking interactions with DFHBI that is complemented by hydrogen bonding of DFHBI to the neighbouring G31. π-π stacking interaction of DFHBI with the UAU cap has a pronounced effect on the absorption spectrum that is further enforced by the π-π interaction of DFHBI with the upper tetrad of the G-quadruplex platform. The prominent effect of the cap as shown in Fig. S7 may explain the finding that mutation of the UAU triplet leads to a significant reduction of both, of the fluorescence yield and of the redshift of its fluorescence excitation spectrum (Mandal et al. 2004). In contrast to the influence of the G4-platform and the UAU cap, H-bonding of DFHBI to the neighbouring G31 has very little effect on the absorption spectrum. H-bonding, however, may well be the predominant binding mode of a multitude of dark complexes.

Warner, K. D., Chen, M. C., Song, W. J., Strack, R. L., Thorn, A., Jaffrey, S. R. & Ferré D'Amaré, A. R. Structural basis for activity of highly efficient RNA mimics of green fluorescent protein. *Nat. Struct. Mol. Biol*. **21,** 658-663 (2014).

Hirata, S. & Head-Gordon, M. Time-dependent density functional theory within the Tamm–Dancoff approximation. *Chem. Phys. Lett.* **314**, 291-299 (1999).

Mandal, D., Tahara, T. & Meech, S.R. Excited state dynamics in the green fluorescent protein chromophore. *J. Phys. Chem. B* **108**, 1102-1108 (2004).

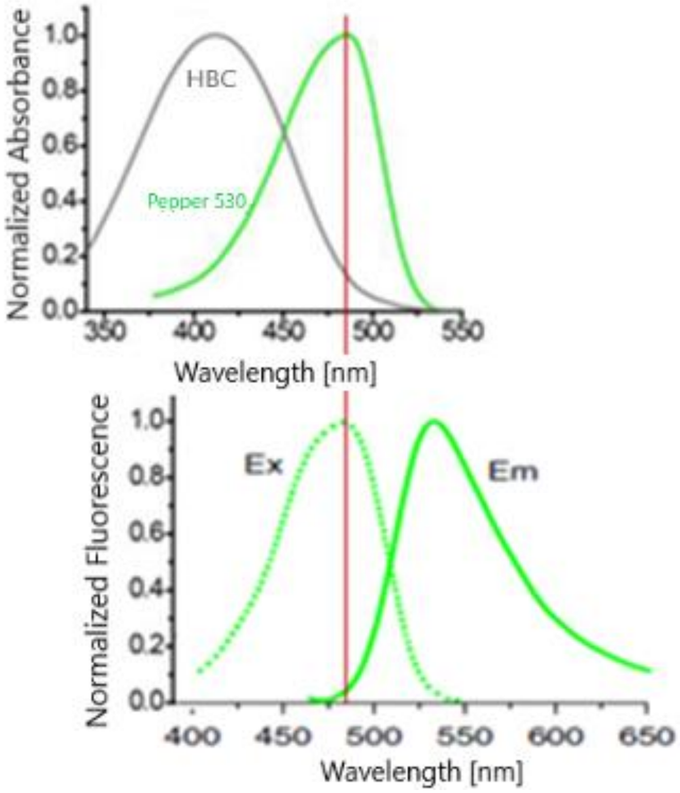

Figure S8. Overlap of absorption and fluorescence excitation spectra of the HBC (4-((2-**H**ydroxyethyl)(methyl) amino)-**B**enzylidene-**C**yanophenyl-acetonitrile in complex with RNA aptamer (Pepper 530).

Top: Fig. S1I and Bottom: Fig. 1e from

Chen et al. Visualizing RNA dynamics in live cells with bright and stable fluorescent RNAs. *Nat. Biotech*. **37**,1287-1293 (2019)

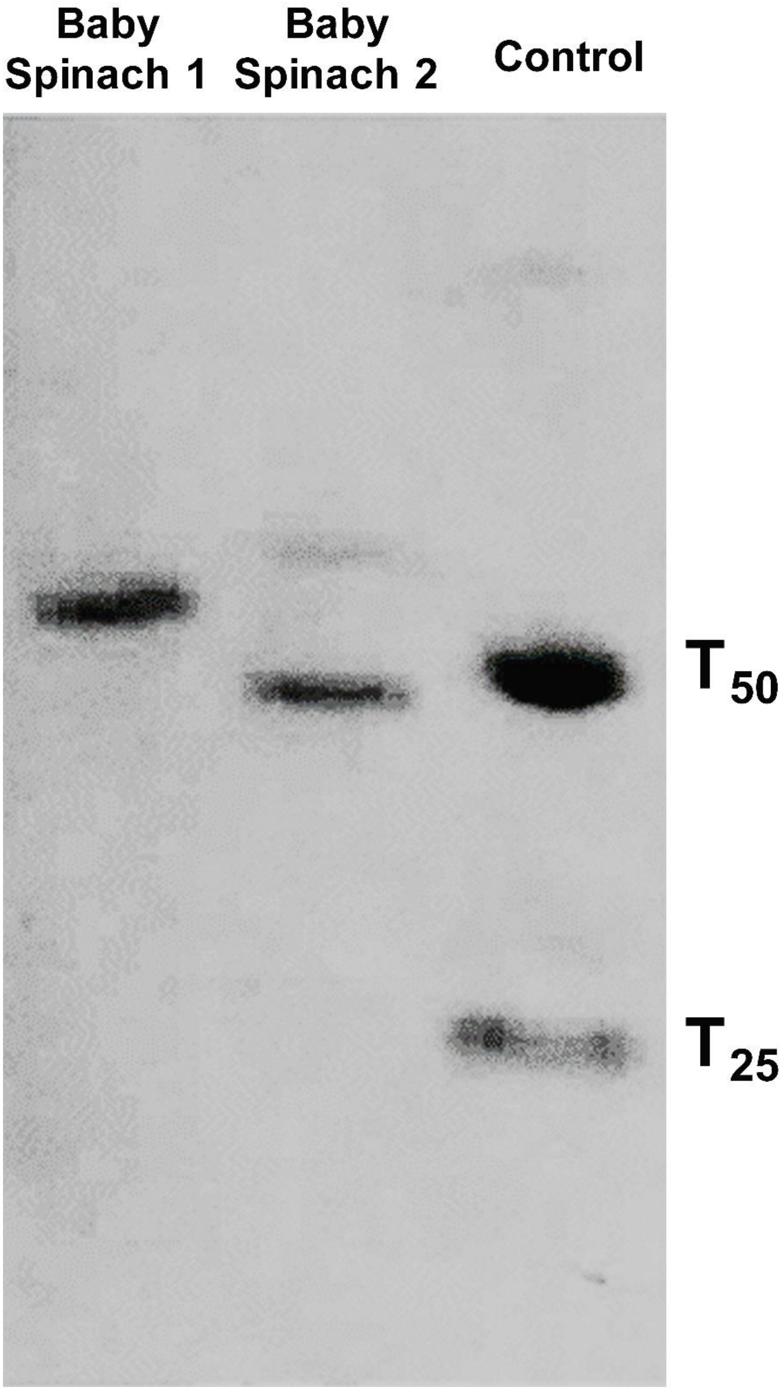

Figure S9. Denaturing urea polyacrylamide gel electrophoresis (Urea-PAGE): The first and second lanes are Baby Spinach RNA sequence at different pH condition – 7.5 (similar to the HEPES buffer condition of the complex formation) and 8.3 (our typical running gel condition). The last land is dT_{50} and dT_{25} marker. RNA concentration is 20 µM. Gel electrophoresis experiments were performed in 20 × 20 cm native gel containing Urea (ultrapure) 40% polyacrylamide solution (29:1) in TBE buffer (pH 8.3). Electrophoresis was run in 1x TAE buffer at 140 V for 6 h at 55 °C by northern blot technique, and the gel was viewed by UV shadowing method (no hybridization with antibodies). The band corresponding to the 51 nt Baby Spinach RNA migrated at closed speed as the DNA oligonucleotides maker dT_{50} and there is no smear band below, indicating the Baby Spinach RNA was intact.

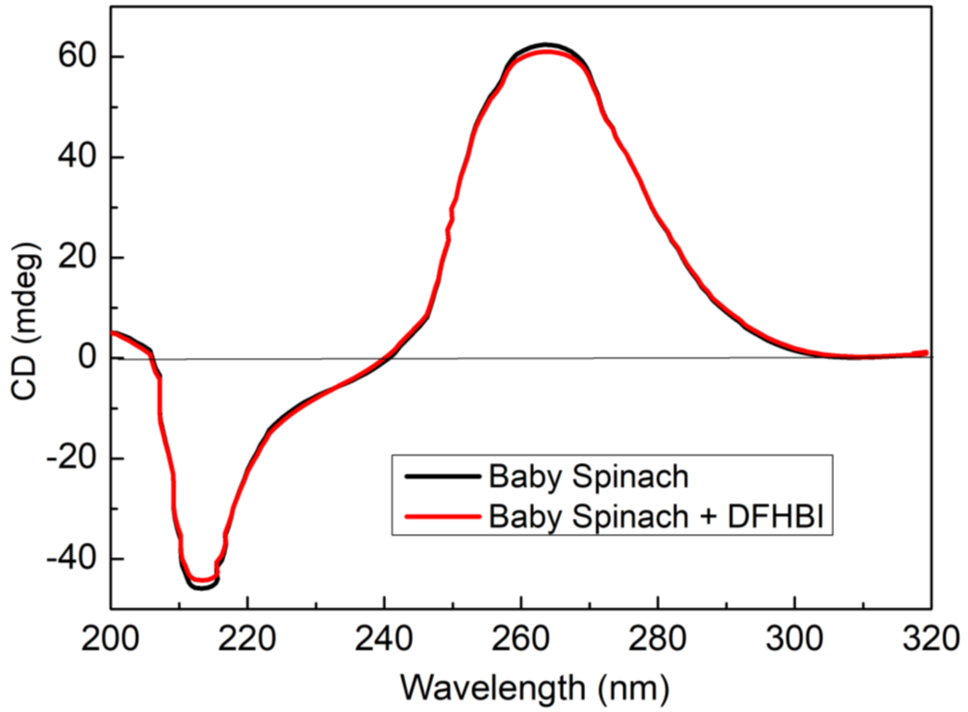

Figure S10. CD spectra of 20 µM Baby Spinach (black) before and (red) after mixing with 2µM DFHBI. Both RNA and DFHBI was prepared in HEPES buffer (10 mM pH 7.5) containing DEPC treated water, 50 mM KCl and 5 mM $MgCl_2$. DFHBI from 200 µM stock was diluted in the same buffer and added into the RNA solution. The mixture was vortexed for 1 min and left in the dark for 2 hours for completion of the binding process, as the same as our typical sample preparation protocol. CD spectra were carried out right after that.

CD spectra were recorded on a Jasco J-815 spectrometer using a standard quartz cuvette with 1-cm path-length. Scans from 220 to 320 nm were acquired with scanning speed of 200 or 500 nm/min, step of 1 nm, and bandwidth of 1 nm.

A positive band at 264 nm confirmed the formation of G-quadplex binding pocket, as previous reported by DasGupta et. al. for G-quadruplex-containing RNAs, including Spinach derivatives. The Identical CD spectra of the Baby Spinach DFHBI confirmed the formation and the intact of the G-quadruplex binding pocket before and after forming the complex.

DasGupta S, Shelke SA, Li N, Piccirilli JA, Guo L, Wang H, Liang H. Spinach RNA aptamer detects lead(II) with high selectivity. *Chem. Commun.* **51**, 9034–9037 (2015).

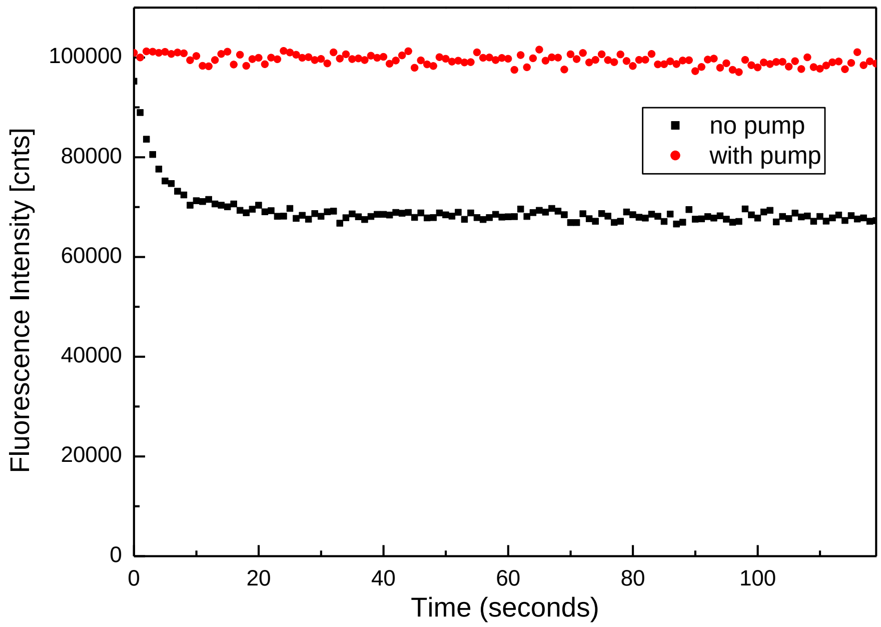

Figure S11. Suppression of photo-bleaching of DFHBI/RNA complex in flow cell. Upon excitation at 416 nm at pump speed of 48 rpm, there is less than 2% decrease of fluorescence.

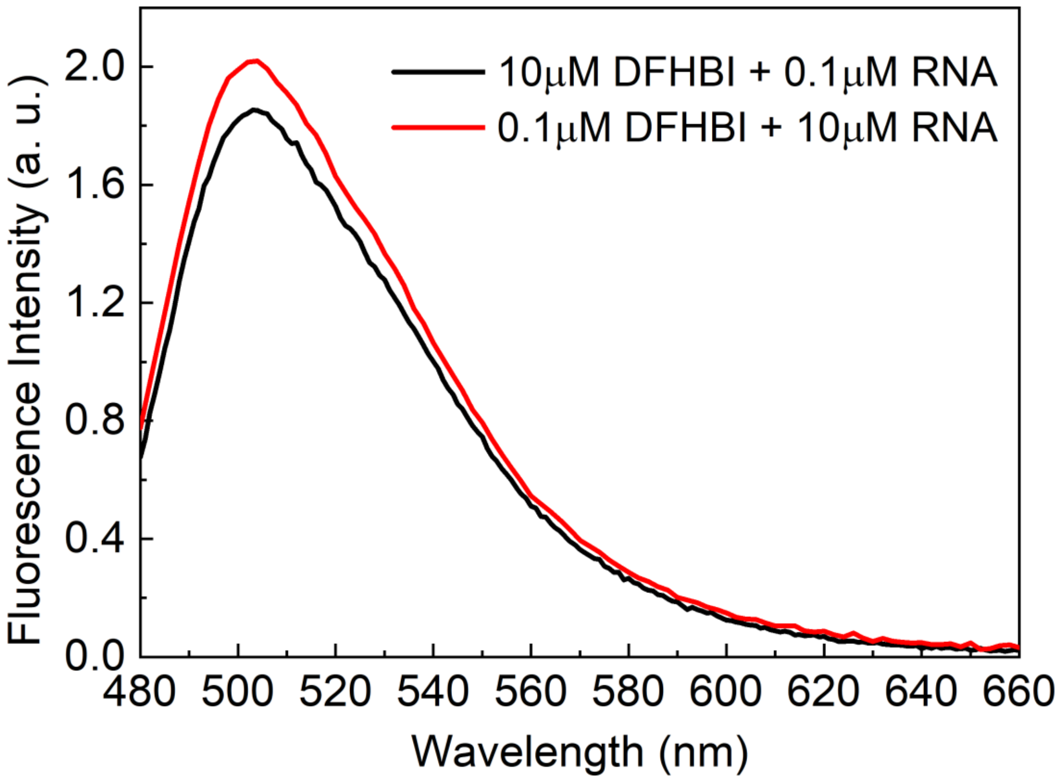

Figure S12. Fluorescence of the mixture (excited at 469 nm) under 2 extreme conditions: (a) DFHBI is in excess relative to the RNA (10 µM DFHBI + 0.1 µM RNA) – black curve and (b) RNA is in excess relative to the DFHBI (0.1µM DFHBI + 10µM RNA) – red curve.
